# Myeloid‑derived suppressor cells as targets of emerging therapies and nanotherapies (Review)

**DOI:** 10.3892/mi.2024.170

**Published:** 2024-06-25

**Authors:** Dileep Kumar, Victor Carlos Da Silva, Natalia Lemos Chaves

**Affiliations:** 1 Department of Genetics and Morphology, Institutes of Biological Sciences, University of Brasilia, Brasilia, DF 70910-900, Brazil; 2 Microscopy and Microanalysis Laboratory, Institutes of Biological Sciences, University of Brasilia, Brasilia, DF 70910-900, Brazil

**Keywords:** breast cancer, immunosuppression, myeloid-derived suppressor cells, tumor microenvironment, immunotherapy, nanotherapy

## Abstract

Breast cancer (BC) is the leading cause of cancer-related mortality among women worldwide. Immunotherapies are a promising approach in cancer treatment, particularly for aggressive forms of BC with high mortality rates. However, the current eligibility for immunotherapy remains limited to a limited fraction of patients with BC. Myeloid-derived suppressor cells (MDSCs), originating from myeloid cells, are known for their dual role in immunosuppression and tumor promotion, significantly affecting patient outcomes by fostering the formation of premetastatic niches. Consequently, targeting MDSCs has emerged as a promising avenue for further exploration in therapeutic interventions. Leveraging nanotechnology-based drug delivery systems, which excel in accumulating drugs within tumors via passive or active targeting mechanisms, are a promising strategy for the use of MDSCs in the treatment of BC. The present review discusses the immunosuppressive functions of MDSCs, their role in BC, and the diverse strategies for targeting them in cancer therapy. Additionally, the present review discusses future advancements in BC treatments focusing on MDSCs. Furthermore, it elucidates the mechanisms underlying MDSC activation, recruitment and differentiation in BC progression, highlighting the clinical characteristics that render MDSCs suitable candidates for the therapy and targeted nanotherapy of BC.

## 1. Introduction

Breast cancer (BC) is the most commonly diagnosed type of cancer among women globally, with >2.3 million cases identified in 2020. In that same year, ~685,000 women succumbed due to BC, making it the leading cause of cancer-related death in women (1,2). BC is classified into four subtypes based on the expression of human epidermal growth factor receptor 2 (HER2/neu), progesterone receptor and estrogen receptor triple-negative breast cancer (TNBC), namely as HER2-positive, Luminal A and Luminal B. The treatment options for BC primarily involve surgical interventions, chemotherapy, radiation therapy, hormonal therapy and immunotherapy (3). It is crucial to tailor the treatment approach to the subtype, stage and specific characteristics of the cancer, alongside considering the overall health and preferences of the patient. During BC treatment, patients commonly face challenges, such as side-effects, including nausea, hair loss, fatigue, neuropathy and an elevated susceptibility to infections. Chemotherapy (4,5) and certain treatments, such as trastuzumab (Herceptin), may provoke adverse reactions with potential cardiac toxicity (6,7). Despite the increasing importance of immunotherapy in the treatment of various types of cancer, its efficacy in BC remains limited, benefiting only a small fraction of patients (8). Moreover, there have been reports of immune-related adverse events linked to the use of immune checkpoint inhibitors, often resembling autoimmune diseases, but with a more acute onset and potential for severe, lasting effects. The complexity and heterogeneity of BC pose significant challenges for immunotherapies, particularly immune checkpoint inhibitors (9,10). Without validated targeted therapies, chemotherapy remains the standard treatment strategy for BC (11). However, alongside known adverse effects, the development of drug resistance is a significant issue, leading to disease progression and increased mortality rates. Recent research efforts have focused on overcoming resistance by exploring combinations of cytotoxic, targeted and immune-based therapies (12). Recognizing the crucial role of the tumor microenvironment (TME) in drug resistance underscores the importance of understanding tumor immunity for developing effective immunotherapeutic strategies against BC.

Myeloid-derived suppressor cells (MDSCs) are critical components of the TME, playing a crucial role in suppressing immune responses in cancer, infections and inflammatory diseases. Originating from immature myeloid cells (IMCs), MDSCs exhibit marked heterogeneity, comprising pathologically activated neutrophils and macrophages (13). Recent investigations underscore their dual function in suppressing antitumor immune responses, while stimulating tumor progression. MDSCs promote tumor angiogenesis, facilitate tumor cell invasion and contribute to the formation of premetastatic niches (14,15). MDSC levels are closely associated with clinical outcomes and therapeutic efficacy in patients with BC (16).

In BC, MDSC accumulation in the TME and peripheral circulation is notable, driven by the modulation of immunosuppressive mechanisms, predominantly through T-cell activation inhibition (17), along with the secretion of multiple cytokines and non-immunosuppressive pathways (18). This collective action promotes tumor growth by enabling tumor angiogenesis, enhancing invasion and metastasis, and modifying the TME to favor tumor progression (19). MDSCs play diverse roles in promoting tumor development by impeding the immune system. Given their pivotal role in subverting the body's antitumor defenses, MDSCs are increasingly recognized as promising targets for therapeutic interventions, including innovative approaches such as nanotechnology.

The present review discusses the immunosuppressive functions of MDSCs, their role in BC, and strategies for targeting them in cancer therapy.

## 2. MDSCs in BC

MDSCs derived from patients with BC exhibit functional and phenotypic similarities to those originating from bone marrow, indicating their myeloid precursor origin (13). These MDSCs are categorized into monocytic (M)-MDSCs (CD11b^+^ Ly6G^-^ Ly6C^high^) and granulocytic (G)-MDSC (CD11b^+^ Ly6G^+^ Ly6C^low^) subpopulations (20,21). In humans, M-MDSCs are characterized by CD11b^+^CD33^+^HLA-DR^-/low^ CD14^+^ CD15^-^ markers, while G-MDSCs express CD11b^+^ CD33^+^ HLA-DR^-/low^ CD14^-^ CD15^+^ (or CD66b^+^) markers (20,22).

MDSC development is regulated by a network of signals that promote the growth of IMCs (23). Various signaling pathways and regulators, such as the signal transducer and activator of transcription (STAT) family, interferon (IFN) regulators, Notch, adenosine receptor A2b and NLRP3, facilitate myelopoiesis, inhibit the maturation and differentiation of progenitor cells, and expand the IMCs. Additionally, signaling pathways and regulators, including NF-κB, STAT1, STAT6, prostaglandin E2 (PGE2), cyclooxygenase-2 and the endoplasmic reticulum stress response contribute to the development of an immunosuppressive phenotype that leads to the pathological activation of immature cells (23,24).

**Figure 1:**
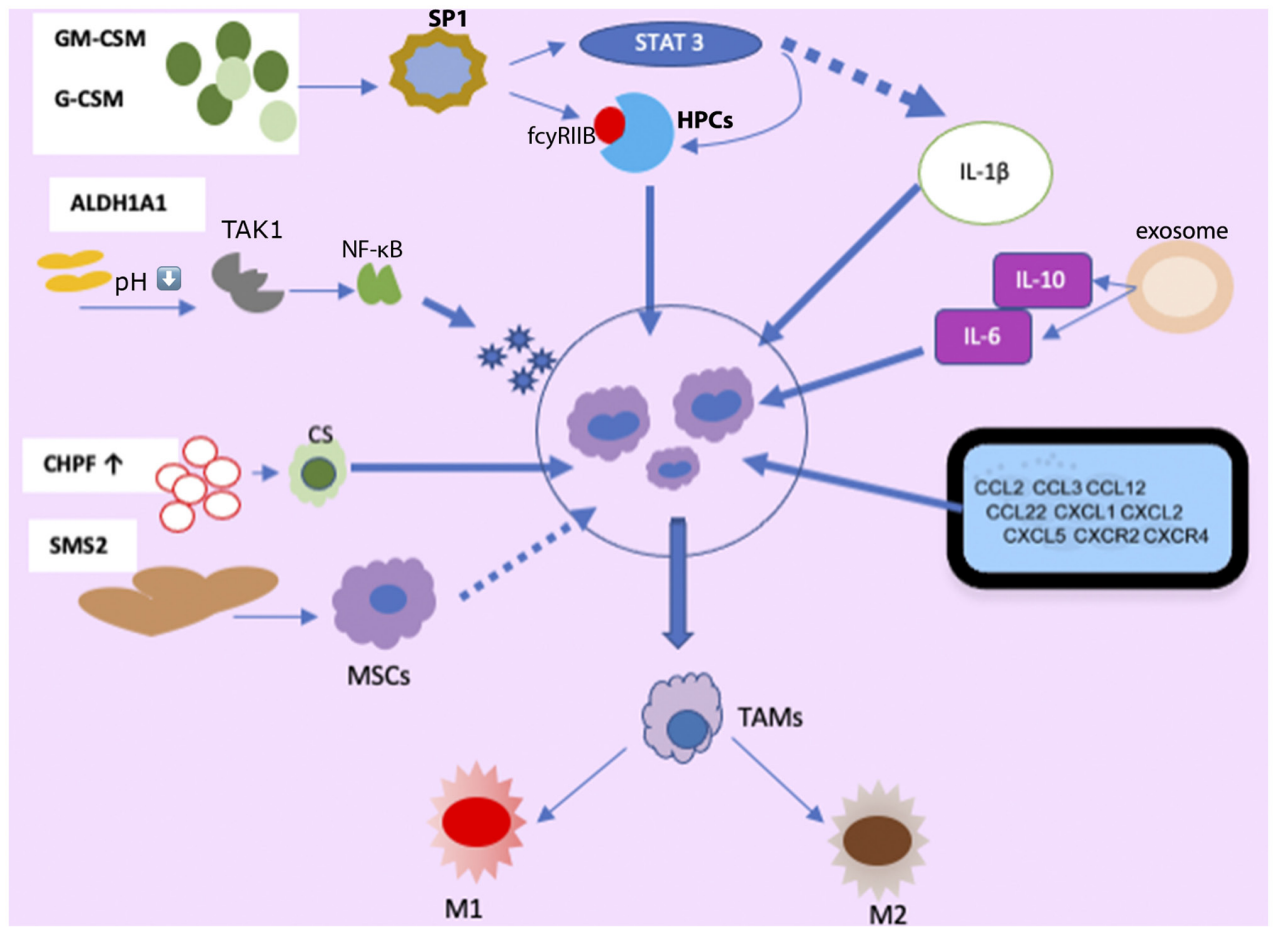
Mechanisms involved in the production, activation, recruitment and differentiation of MDSC in BC. MDSCs, myeloid-derived suppressor cells; HPCs, hematopoietic progenitor cells; SP1, specific protein 1; GM-CS, granulocyte-macrophage colony-stimulating factor; G-CSF, granulocyte colony-stimulating factor; ALDH1A1, aldehyde dehydrogenase 1A1; TAK1, TGF-β-activated kinase 1; CS, chondroitin sulfate; CHPF, chondroitin polymerase factor; SMS2, sphingosine synthase 2; MSCs, mesenchymal stem cells; TAMs, tumor-associated macrophages; M1, M1 macrophages; M2, M2 macrophages.

Factors, such as granulocyte colony-stimulating factor (G-CSF), granulocyte-macrophage colony-stimulating factor (GM-CSF) (24-26), vascular endothelial growth factor (VEGF), PGE2, interleukin (ILs)s [IL-1 (27-29), IL-6, IL-13, IL-17, IL-20, IL-33, IL-34 (30-35)], macrophage migration inhibitory factor (MIF) (36), along with microRNAs (miRNAs/miRs), contribute to MDSC amplification and activation in BC (37). Of note, patients with BC with MDSCs often exhibit higher levels of psychological stress, likely influenced by stress-related hormones and cytokines, such as IL-1Ra, IFN-γ-induced protein 10, G-CSF and IL-6, further stimulating MDSC production and accumulation (38) (Fig. 1).

G-CSF and GM-CSF, derived from tumor cells, play pivotal roles in MDSC accumulation (23,25). MDSCs originate from immature, multipotent hematopoietic progenitor cells (HPCs) and respond to signals from the host and tumor cells, particularly through the secretion of GM-CSF, and are subsequently recruited to the TME (39).

Among the receptors implicated in MDSC regulation, FC gamma receptor IIB (FCγRIIB/CD32B) is the sole inhibitory member expressed on B-cells, macrophages, dendritic cells (DCs) and granulocytes, with an upregulation of its expression observed in tumor-infiltrating MDSCs (40). GM-CSF increases FcγRIIB expression in HPCs by activating specificity protein family 1 (SP1) transcription factors, which bind to GC-rich motifs, thereby regulating the expression of genes involved in proliferation, apoptosis, differentiation, and immune responses. The inhibition of SP1 dampens MDSC differentiation and infiltration into the TME. However, when SP1 and FCγRIB are activated, they promote MDSC generation from HPCs via STAT3, a member of the STAT family of transcription factors activated by tyrosine kinases in response to various cytokines and growth factor receptors (41,42). Consequently, tumor cell-induced activation of GM-CSF-driven Sp1 and STAT3 cooperatively trigger the expression of target genes, facilitating the immunosuppressive functions of MDSCs. Moreover, chondroitin polymerase factor, frequently overexpressed in BC tissues, enhances G-CSF binding to cell surface chondroitin sulfate, thereby promoting MDSC accumulation (43).

The enzymatic activity of aldehyde dehydrogenase 1A1 (ALDH1A1) is crucial in reducing intracellular pH in BC cells. This condition triggers increased TGF-β-activated kinase 1 (TAK1) phosphorylation, subsequently activating the NF-κB pathway. Consequently, GM-CSF secretion is induced, amplifying MDSCs and fostering BC progression (44). Exosomes released by 4T1 (syngeneic cell lines derived tumor models) cells contain IL-6 and IL-10, which enhance MDSC stimulation and proliferation by promoting STAT3 phosphorylation in myeloid cells. This diminishes myeloid proliferation and death, expediting differentiation into MDSCs (37).

The study by Jiang *et al* (45) revealed that tumor exosome-secreted miR-9 and miR-181a targeted suppressor of cytokine signaling protein 3 (SOCS3) and separately activated STAT3 (PIAS3), triggering the JAK/STAT signaling cascade. Prolonged SOCS3 suppression and abnormal JAK/STAT pathway upregulation lead to early-stage MDSC accumulation (46). A number of molecules in signaling pathways form intricate regulatory loops. In BC cells, the mammalian target of the rapamycin pathway induces MDSC accumulation by modulating G-CSF expression. MDSCs reciprocally increase tumor-initiating cell frequency by activating the Notch signaling pathway in tumor cells, which secretes G-CSF, establishing a feed-forward loop promoting MDSC expansion (47). Autocrine secretion of GM-CSF and IL-33 in the TME sustains MDSC viability by inhibiting apoptosis, promoting a positive feedback loop that induces MDSC accumulation (34).

### Mechanisms of the recruitment of MDSCs in BC

Several factors, including chemokines, cytokines, and complements produced by both tumor and normal cells, induce the recruitment of MDSCs into tumor tissue (48). Among these, lung fibroblasts secrete chemokine (C-X-C motif) ligand (CXCL)1, which promotes an immunosuppressive lung microenvironment by attracting granulocytic MDSCs and facilitating the formation of BC metastatic niches in the anterior lung (49).

BC exosomes carry elevated levels of miR-200b-3p, which are taken up by alveolar epithelial type II cells, directly impacting phosphatase and tensin homolog (PTEN). PTEN suppression activates the AKT/NF-κB-p65 pathway, increasing chemokine (C-C motif) ligand 2 (CCL2) expression and attracting MDSCs, ultimately promoting lung metastasis in BC (50). Endoplasmic reticulum oxireductin 1a, a disulfide oxidase located in the endoplasmic reticulum and closely associated with tumors, participates in the oxidative folding process, generating and attracting granulocytic MDSCs, and contributes to G-CSF, CXCL1 and CXCL2 production (51). TGF-β1 upregulates miR-494 levels in MDSCs, enhancing MDSC movement via CXCR4(52). The transcription factor, ΔNp63, directly regulates CXCL2 and CCL22, facilitating MDSC recruitment in TNBC (53).

Liver cells contribute to MDSC recruitment at specific sites by producing S100A8, facilitating BC metastasis (54). Chen *et al* (55) found that VEGF-C produced by breast cancer cells was responsible for increasing the levels of chemokines produced by lymphatic endothelial cells (LECs). This, in turn, helped recruit MDSCs to the TME and lymph nodes (LNs) through the CXCR2 pathway (55). Evidence suggests that in the presence of 4T1 cells (breast cancer cell line derived from the mammary gland tissue of a mouse BALB/c strain), interstitial fluid migration alongside LECs aids MDSC dissemination. Moreover, reduced levels of vascular VEGFR3 decrease the flow response in MDSCs and 4T1 cells (56). The acetylation of the SMAD3 protein, dependent on the epigenetic regulator KAT6A, contributes to MDSC recruitment and TNBC metastasis through epigenetic regulation (57).

The activation of the complement system, particularly through C5a signaling, plays a pivotal role in recruiting MDSCs into the TME and suppressing CD8^+^ T cell-mediated tumor elimination. Consequently, lung angiogenesis is fostered in tumor-bearing mice, facilitating BC metastasis to this organ (58). Cheng *et al* (59) demonstrated that periodontal inflammation (PI) activation enhances the expression of chemokines such as CCL5, CXCL12, CCL2 and CCL5, which recruit MDSCs, thereby promoting the establishment of premetastatic niches at sites of inflammation. MDSCs exhibit diverse differentiation pathways regulated by various transcription factors, as depicted in Fig. 1. They can differentiate into tumor-associated macrophages (TAMs) and dendritic cells, which further stimulate the production of inflammatory DCs (60).

During BC progression, MDSCs transition into TAMs. Under environmental pressures, such as hypoxia, M-MDSCs differentiate into TAMs upon migration to specific tissues. TAMs, in turn, can adopt either the M1 phenotype, characterized by pro-inflammatory and antitumor properties, or the M2 phenotype, which exhibits protumor functions, in response to stimuli such as lipopolysaccharide (LPS), TNF-α and IFN-γ (61).

Macrophages and MDSCs are ubiquitous in the majority of solid tumors, driving immune suppression and inflammation (61). Their interaction increases IL-10 production by MDSCs and decreases IL-12 production by macrophages, polarizing the immune system toward a type 2 protumor environment (62). IL-33 is known for its stimulatory effects on myeloid and lymphoid cells, promoting the production of type 2 cytokines. The activation of ST2 in type 2 innate lymphoid cells (ILC2) triggers the release of type 2 cytokines IL-33 and IL-13(63). ILC2 are tissue-resident lymphocytes with various functional roles in mucosal immunity. In tumor immunology, ILC2 is crucial in DC recruitment via CCL5 production and activation through IL-9 and IL-13 secretion (64). IL-33 further stimulates ST2^+^ regulatory T-cells (Tregs) and amphiregulin (AREG) expression, enhancing immune regulatory functions and tissue repair (65). The activation of ILC2 through IL-33 supports type 2 immune responses and M2 reparative macrophages. ST2 is also expressed in myeloid-derived antigen-presenting cells, such as macrophages and CD11b^+^ CD11c^+^ DCs (66). Moreover, IL-33 triggers IL-2 release and fosters Treg cell expansion. The type, density and spatial distribution of these IL-33-modulated immune cells within tumors profoundly influence tumor behavior (67). Thus, the cytokine IL-33 stimulates the upregulation of IL-13 while concurrently suppressing IL-12 levels. This immune profile underlies the negative impact of M2 macrophages and Th2 cell polarization within the TME on antitumor immunity (34).

TAMs are the most abundant immune cells involved in regulating breast cancer progression. The TME contains many immunosuppressive cells (68). Macrophages exhibit heterogeneity, with at least two functionally distinct subtypes responding to different stimuli: classically activated M1 macrophages and alternatively activated M2 macrophages (69). M1 macrophages eliminate tumors directly by recognizing and phagocytizing cancer cells and indirectly by producing pro-inflammatory cytokines, such as IFN-γ and IL-12. Conversely, in the context of tumor development, M2 macrophages are regarded as ‘tumor promoters’. They facilitate cancer progression, promote tumor cell metastasis and angiogenesis, regulate energy metabolism and aid in immune system evasion (70). During tumor progression, M2 macrophages become more prevalent, eventually dominating the TAM population in the TME. The underlying mechanism suggests that TAMs promote and sustain cancer stem cells, thereby supporting tumor growth and self-renewal. Various cytokines and signaling pathways within the TME influence the polarization of macrophages into M1 or M2 types. When IL-4 binds to its receptor, it can promote the phosphorylation of STAT6, leading to the polarization of M2-like macrophages through the JAK/STAT6 signaling pathway (68,71). Phosphorylated STAT6 can bind to Krüppel-like factor 4 (KLF4) and peroxisome proliferator-activated receptor γ, further promoting this polarization. Furthermore, various signals, including TGF-β, IL-10, bone morphogenetic protein-7 and IL-4 itself, induce M2 polarization through the PI3K/Akt signaling pathway. The complex formed by CCAAT/enhancer binding protein α and KLF6 is also associated with the switch from the M1 to the M2 phenotype (71,72). The progression of BC can also be observed through the promotion of monocytic MDSC differentiation into immunosuppressive M2-polarized macrophages, facilitated by both sphingosine synthase 2 and exosomes secreted by mesenchymal stem cells (61,73). Doxorubicin (DOX)-resistant tumor cells release PGE2, activating the EP2-EP4/cAMP/PKA signaling cascade in MDSCs, fostering proliferation and an M2 polarized phenotype shift. This polarization is mediated through miR-10a induction (27). Studies have shown that natural killer (NK) T-cells (NKT cells) help transform CD11b^+^ HLA-DR MDSCs into CD11b low HLA-DR DCs (74).

### Immunosuppressive effects of MDSCs on BC progression

**Figure 2:**
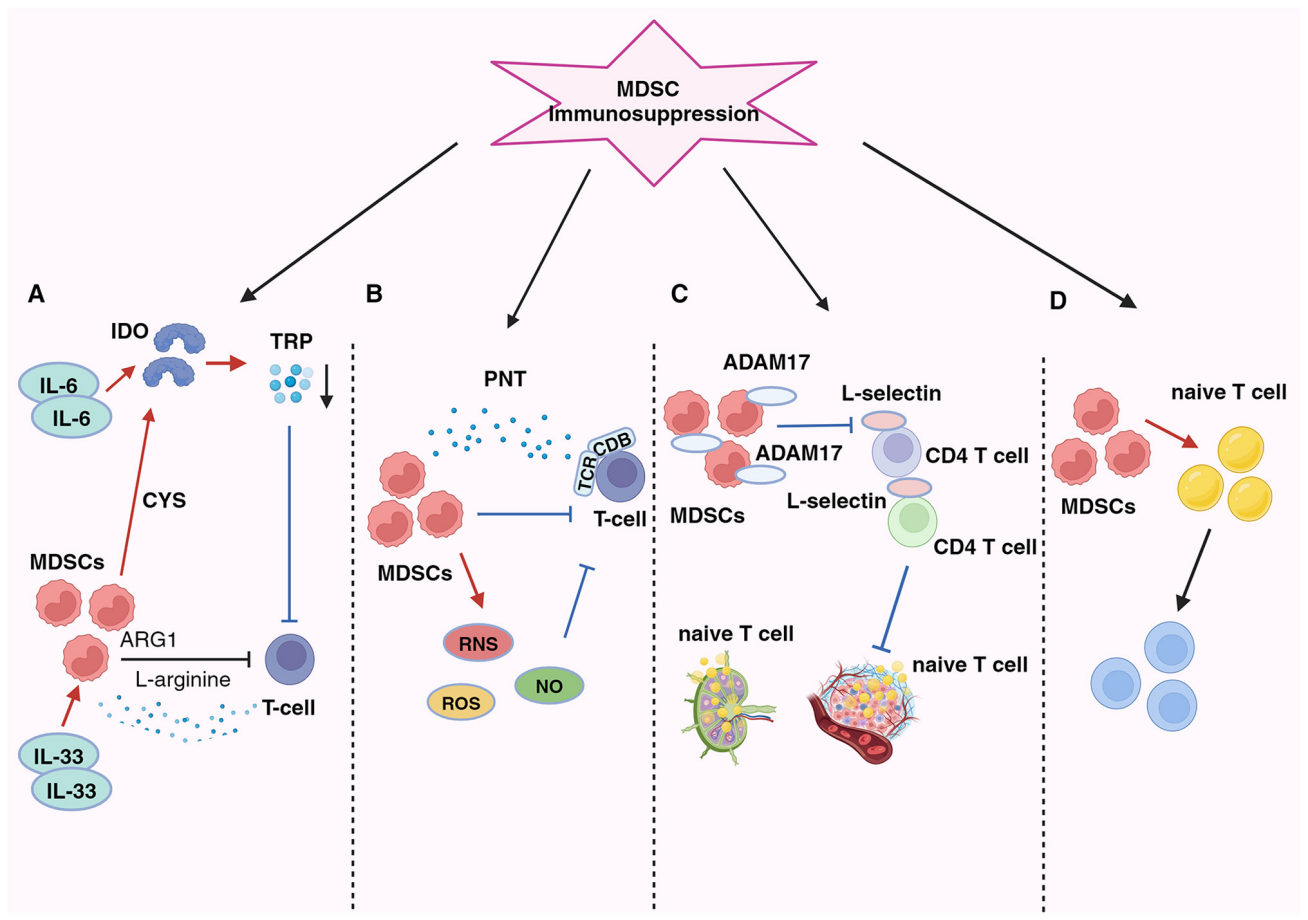
Presence of MDSCs in BC and their diverse immunosuppressive effects (A) MDSCs suppress T-cells by activating IDO, resulting in the depletion of essential nutrients, such as ARG1, TRP and cysteine (CYS). (B) MDSCs induce oxidative stress by generating ROS, RNS and NO, leading to T-cell suppression. (C) MDSCs impede lymphocyte migration through direct physical contact and the expression of ADAM17. (D) MDSCs disrupt the conversion of naïve CD4^+^ T-cells into Tregs. MDSCs, myeloid-derived suppressor cells; PNT, peroxynitrite; TCR, T-cell receptor; IDO, indoleamine 2,3-dioxygenase; ARG1, arginase 1; TRP, tryptophan; CYS, cysteine; ROS, reactive oxygen species; RNS, reactive nitrogen species; NO, nitric oxide; ADAM17, a disintegrin and metalloproteinase domain 17.

Fig. 2 shows how MDSCs significantly impede the activity of tumor-fighting T- and B-cells within the TME, particularly cytotoxic T-lymphocytes (CTLs) and pro-inflammatory cells, such as NK cells. Additionally, MDSCs can promote cancer progression by inducing the generation of Tregs and T-helper 17 (Th17) cells, thereby altering the local environment to promote tumor growth and enable immune evasion (75).

The primary immunosuppressive mechanism in the BC microenvironment involves the inhibition of T-cell function, which is the main trigger process involving the depletion of vital nutrients (Fig. 2A) (76). MDSCs exert inhibitory effects by activating indoleamine 2,3-dioxygenase (IDO), reducing local tryptophan availability, and generating cytotoxic metabolites, such as kynurenine in the TME and lymphatic drainage regions. This leads to an increase in Tregs, the inhibition of immune responses against antigens and the suppression of tumor-specific CTLs (77). The activation of STAT3-dependent NF-κB by IL-6 is responsible for maintaining IDO overexpression (30,77). Additionally, MDSCs enhance the suppressive action of IL-33 on T-cells by depleting L-arginine via arginase 1 (ARG1) (33,75). Furthermore, MDSCs consume cysteine, essential for T-cell activation and optimal function, leading to its depletion and failure to replenish in their environment (78). The generation of oxidative stress is a key factor in the second mechanism (Fig. 2B).

By producing reactive oxygen species, reactive nitrogen species and nitric oxide (NO), MDSCs suppress T-cells in the TME. MDSCs induce immune tolerance by T-cell receptor and CD8^+^ surface modifications and generate the free radical peroxynitrite (79). According to Stiff *et al* (80), MDSC-generated NO also disrupts Fc receptor-mediated NK cell activity, reducing monoclonal antibody efficacy and impeding the immune response against tumors. The third mechanism occurs by preventing lymphocyte migration (Fig. 2C). MDSCs reduce the immune response in peripheral lymphoid organs and accumulate in sentinel LNs, where they impede CD3/CD28-induced T-cell proliferation through contact-dependent mechanisms. This process supports tumor progression and metastasis (80,81). Hanson *et al* (82) linked decreased L-selectin expression on CD4^+^ and CD8^+^ T-cells with a disintegrin and metalloproteinase domain 17 generation on the plasma membrane. Consequently, MDSCs in BC hinder immature T-cell activation and migration into LNs and their subsequent transport to tumors, ultimately compromising the immune system's capacity to combat tumors (82).

The fourth aspect pertains to the expansion and activation of Tregs, facilitated by MDSCs via promoting their proliferation and differentiation of naïve CD4^+^ T cells (Fig. 2D). Although this pathway mechanism is not yet fully understood, it is known that Tregs can infiltrate tumors and play a crucial role in the antitumor immunosuppressive response. IL-34 triggers the conversion of bone marrow stem cells into monocytic MDSCs, indirectly suppressing the immune response by fostering Treg attraction via CCL22 secretion in the TME, thus contributing to chemotherapy resistance (35). Additionally, BC-induced MDSCs can stimulate effector T-cells to transition into Tregs through the IDO mechanism (77). MDSCs suppress T-cell activation, and once activated, T-cells trigger MDSC apoptosis via the Fas-FasL pathway (83).

MDSCs in BC employ contact-dependent mechanisms and indirect means, releasing NO, ARG and IL-1 to suppress the response of B-cells against tumors (84). Additionally, MDSCs can transform ordinary B-cells into specialized immunomodulatory B-cell (Breg) phenotypes, which efficiently suppress T-cell responses (85). Furthermore, various mediators in the TME, including LPS, can induce programmed cell death protein 1 (PD-1) expression in MDSCs in BC (86). By activating the PI3K/PKB/NF-κB signaling pathway in B-cells, MDSCs can enhance immune evasion mediated by PD-1/PD-L1 Bregs through the PD-1 pathway (87,88).

BC cells cultured under hypoxic conditions secrete various cytokines, including monocyte chemotactic protein-1, which triggers MDSC recruitment and suppresses the cytotoxic activity of NK cells. These mechanisms collectively contribute to cancer metastasis facilitation (89). NKT cells can restore suppressed T-cell function by converting CD11b^+^ HLA-DR MDSCs into CD11b low HLA-DR DCs through an NKG2D-dependent signaling mechanism (74). The function of MDSCs is further influenced by C5aR signaling, regulating CD4^+^ T-cell polarization towards the Th2 phenotype in the lungs of tumor-bearing mouse (58). The administration of DOX increases miR-126 exosomes derived from MDSCs, thereby suppressing T-cell functionality, inhibiting Th1 cell activation, and initiating Th2 cell responses in mouse lungs (90).

### Clinical aspects of MDSCs related to BC

MDSC levels are associated with the progression of BC, typically showing higher levels in more advanced cancer stages (22). Additionally, surgical stress induced by the excised primary tumor environment can trigger the recruitment of MDSCs to lung and tumor tissues, underscoring the importance of monitoring MDSC levels (91). Data indicate variations in MDSC levels among patients undergoing antitumor treatment, potentially reflecting individual responses to therapy. In neoadjuvant therapy, circulating granulocytic MDSCs may initially increase, decrease with DOX and cyclophosphamide administration, and return close to baseline levels during paclitaxel treatment. Conversely, in metastatic or recurrent BC, monocytic MDSCs undergo significant expansion in the peripheral circulation, being associated with increased degrees of metastases to LNs and other organs (92). Therefore, tracking M-MDSC levels in patients with BC may be a valuable biomarker for monitoring cancer progression and treatment response.

### BC treatments targeting MDSCs

**Figure 3:**
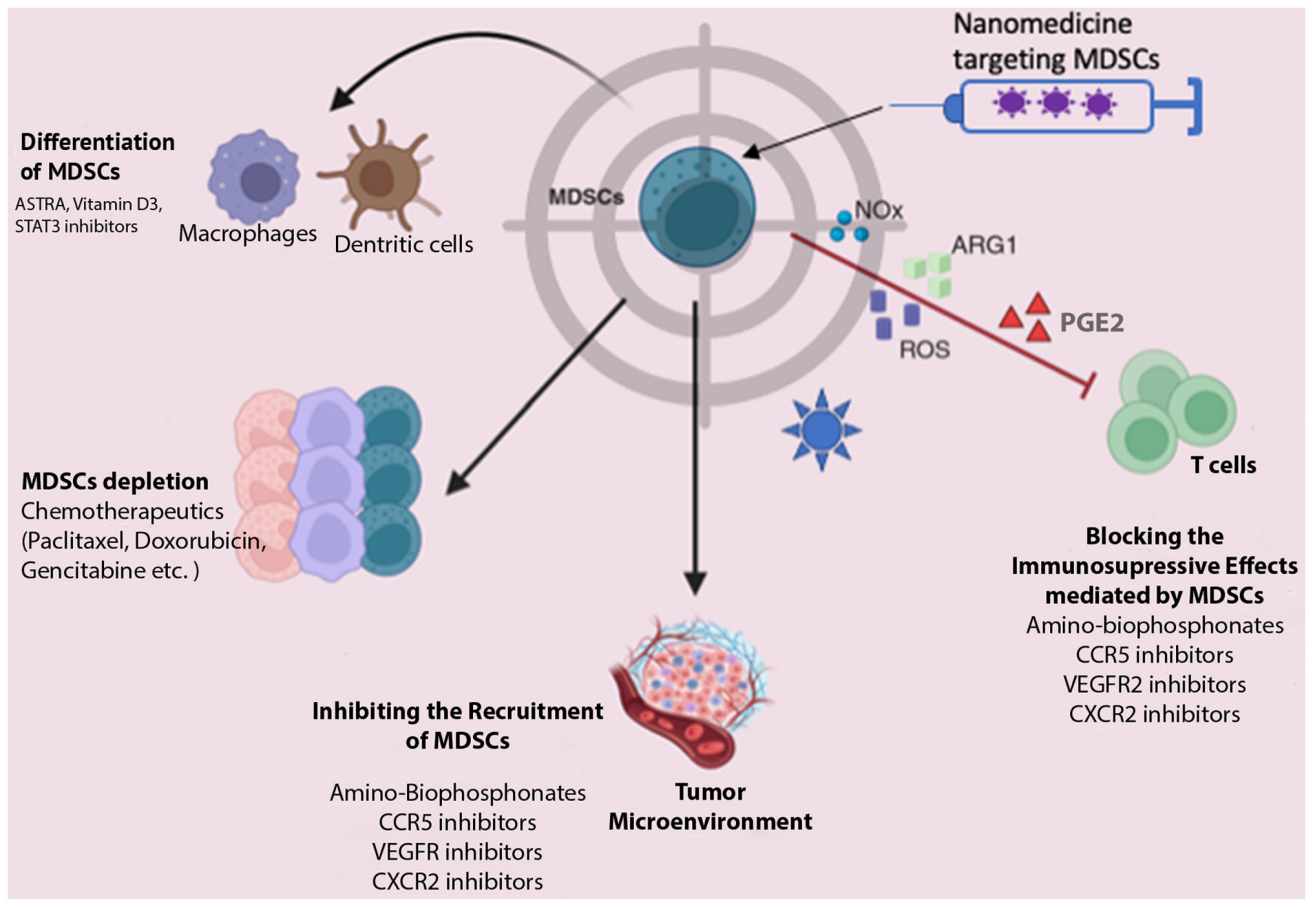
Key strategies for targeting MDSCs that can be exploited by nanotechnology independently or in conjunction with pharmaceutical agents. i) Depleting MDSC populations. ii) Preventing recruitment and migration of MDSCs to the tumor microenviroment. iii) Weakening immunosuppressive functions of MDSCs by lowering the expression of ARG1, inducible nitric oxide synthase, COX-2 and minimizing ROS generation. iv) Stimulating the differentiation of MDSCs into non-suppressive mature myeloid cells such as macrophages and dendritic cells. Illustrative examples are provided for each therapeutic tactic. MDSCs, myeloid-derived suppressor cells; ARG1, arginase 1; ROS, reactive oxygen species; NOx, NADPH oxidase enzyme; PGE2, prostaglandin E2.

MDSCs play a pivotal role in BC progression and are intricately linked to tumor immune evasion. Consequently, MDSCs are a promising target for tumor immunotherapy, primarily aimed at enhancing host immunity. Currently, therapeutic strategies targeting MDSCs encompass four main approaches, as illustrated in Fig. 3: MDSC depletion, the blockade of MDSC recruitment, the suppression of MDSC immunosuppressive function and the induction of MDSC differentiation into a non-suppressive immune state (93-95).

While no specific selective inhibitors of MDSCs have been identified to date, at least to the best of our knowledge, several existing drugs exert indirect effects on MDSCs. For instance, DNA methyltransferase inhibitors and histone deacetylase (HDAC) inhibitors modulate systemic and intratumoral MDSCs, enhancing the long-term response to immunotherapy (96). Other drugs with the potential to suppress or deplete MDSCs and consequently enhance immunotherapy efficacy include gemcitabine (97), DOX (98) and 5-fluorouracil (99). Additionally, combined therapeutic strategies are under investigation, such as the combination of Romidepsin (an HDAC inhibitor) with cisplatin and nivolumab in TNBC (NCT02393794) (100) and IPI-549 (an inhibitor of PI3Kδ and PI3Kγ isoforms that decreases MDSCs and enhances anti-PD-1 efficacy) with nivolumab in solid tumors (101).

Curcumin is known for its antitumor properties, primarily attributed to its inhibition of IL-6. However, research has revealed that its mechanism also entails the inhibition of MDSCs in both blood and tissues, thus impeding tumor growth (102). Numerous preclinical and clinical studies are also dedicated to exploring strategies for promoting MDSC maturation (103-105). Sulforaphane, an inhibitor of the inflammatory cytokine MIF, disrupts its protumor functions, including the facilitation of MDSC differentiation in the TME. *In vitro* research has shown that MIF inhibitors, such as sulforaphane inhibit the accumulation of MDSCs in the TME, blocking their differentiation and restoring antitumor immunological activity (36). Silibinin modulates CCR2 expression in MDSCs, resulting in decreased MDSC accumulation in blood and tumor tissue (106). NG-monomethyl-L-arginine acetate, an inducible NO synthase inhibitor, blocks MDSC differentiation into osteoclasts, potentially preventing MDSC-mediated BC bone metastasis and associated bone degradation (107). Activated T-cells (ATCs) combined with bispecific anti-CD3 x anti-Her2/neu antibodies (aATCs) effectively modulate MDSCs via INF and IL-2, suppressing their activity and inhibiting tumor growth (108). According to Thakur *et al* (109), aATCs suppress the actions and functions of MDSCs (via IFN and IL-2) and effectively inhibit tumor growth and Treg differentiation.

Therapeutic combinations, such as adoptive cell therapy involving sensitized immune and tumor cells reprogrammed with CD25^+^ NKT, NK and memory T-cells, have achieved the immunosuppression of MDSCs (110). In other strategies tested in preclinical models, a vaccine composed of *Listeria monocytogenes* has been investigated. When MDSCs are infected with a highly attenuated bacterium, *Listeria monocytogenes* (Listeria^at^), their immunosuppressive function is altered. Moreover, Listeriaat-infected MDSCs, which primarily deliver Listeriaat to the microenvironment of metastases and primary tumors, spread from MDSCs to tumor cells. Consequently, Listeriaat immunotherapy significantly reduces the population of MDSCs and can convert MDSCs into an immunostimulatory phenotype that produces IL-12, while concurrently reducing metastasis and tumor growth (111).

## 3. Potential nanotechnology-based therapeutics through MDSC targeting

Nanotechnology-based drug delivery systems are the emerging approaches in cancer therapy, characterized by intensive exploration (112,113). Despite the biological and functional barriers posed by the TME, nanoparticles (NPs) have demonstrated efficacy in enhancing intertumoral drug accumulation through passive or active targeting mechanisms. This optimized biodistribution reduces side-effects and increases therapeutic benefits (114,115). While drug delivery platforms targeting MDSCs in cancer treatment (Fig. 3) are relatively new, their potential is promising, particularly in BC.

As examples of successful results, Zhang *et al* (116) used ursolic acid, a natural pentacyclic triterpenoid known for its antifungal, antibacterial and recently discovered immunomodulatory properties, encapsulated within liposomes to modulate the TME. Following five administrations, treatment with this liposomal formulation led to a significant reduction in biomarker levels across the bloodstream, spleen, and tumor sites. This was coupled with enhanced cytotoxic T-cell activity and consequent reductions in tumor volumes (116). Chen *et al* (117) used gemcitabine-loaded nanocages in combination with IDO-targeted small interfering RNA and PD-L1 antibody designed on the nanocarrier surface. This combinatorial approach aims to improve immunosuppression and overall treatment outcomes in patients with TNBC. The administration of these tri-loaded nanocarriers (GSZMP) in TNBC mice was shown to result in a significant decrease in MDSC proportions compared to the controls, accompanied by increased T-cell infiltration. Additionally, the GSZMP group exhibited significant tumor volume reduction and increased survival rates compared to the control group (118).

Using this targeted delivery strategy to the tumor site, a nanoparticle formulation comprising c-peptide (RGDfk) in low molecular weight heparin-retinoic acid (LMWH-ATRA) micelles loaded with DOX and the immune adjuvant α-galactosyl ceramide (αGC) (RLA/DOX/αGC NP) was proposed. The hydrophilic segment of LMWH selectively bound to P-selectin present on vascular endothelial cells impedes the recruitment of MDSCs. The hydrophobic ATRA segment facilitated MDSC depletion, inducing their differentiation. This multidimensional approach effectively modulated MDSCs, significantly improving the inflammatory and immunosuppressive microenvironment in the lungs and tumor sites while inhibiting NPM formation. The micelles exhibited synergistic effects with other components in their composition (notably αGC), enhancing overall antitumor immunity. Thus, this formulation is a promising therapeutic avenue for addressing BC and lung metastases (119).

## 4. Conclusions and future perspectives

There is evidence to suggest a heightened prevalence of MDSCs in patients with BC, indicating their crucial role in the immune-resistant characteristics of the disease. Given the diverse nature of MDSCs, there is a pressing need for assays that can accurately identify MDSC subtypes in patients with BC. It is imperative to evaluate MDSC levels in both peripheral blood and the TME across various stages and subtypes of BC. Such assessments would provide insight into MDSC generation, expansion, and their function in peripheral blood and the TME, thereby elucidating the connection between MDSCs and the advancement of BC stages. The present review aimed to facilitate the practical application of these research findings and lay the foundation for the diagnosis and treatment of MDSC-related aspects of BC. MDSCs are pivotal in advancing tumor growth and metastasis through complex mechanisms.

Immunotherapy is a promising therapeutic approach in cancer treatment, demonstrating increased survival rates in preclinical models and clinical settings. Until recently, immunotherapy was not considered a viable option for BC treatment as BC was long-viewed as a poorly immunogenic tumor type (120). However, increasing evidence in recent years indicating immunogenic activity across various BC subtypes has shifted this paradigm, highlighting immunotherapy as an increasingly important tool in BC treatment (121).

Clinical trials are underway to combine immunotherapy with other therapeutic modalities in BC to target MDSCs (109,110). This approach is justified as MDSCs are essential in the BC microenvironment, promoting tumor growth and metastasis. Thus, MDSC-targeted therapies are promising as potential treatments in clinical settings. Proposed strategies to inhibit MDSCs include promoting differentiation, modulating production flexibility, initiating recruitment in peripheral organs, and direct elimination, aiming to circumvent the strong toxicity and side effects associated with traditional non-specific and sometimes ineffective chemotherapy.

Several therapies targeting MDSCs, either as standalone immunotherapies or combined with standard methods, such as chemotherapy and radiation, are undergoing preclinical trials to enhance their antitumor capabilities. It is hoped that a more in-depth understanding of the clinical significance of MDSCs will prompt the development of MDSC-focused treatments, ultimately improving the outcomes of patients with BC. The TME and MDSCs play pivotal roles in tumor growth and survival, with their influence particularly pronounced in TNBC. Emerging findings suggest that targeting MDSCs may be a promising alternative therapeutic approach, particularly in immunotherapy, reshaping the immunosuppressive microenvironment and enhancing the efficacy of cancer immunotherapy. In this context, nanotechnology is a valuable tool. With its controlled drug release capabilities and increased tumor accumulation, nanoparticles exhibit potential for cancer treatment. Numerous studies on BC mouse models have shown promising results with nanoparticle usage, including significant tumor shrinkage and changes in the TME components. However, the complexity of the microenvironment poses challenges in manipulating it because changes to one cellular component can lead to cascading effects on others. Despite the great promise of incorporating nanotechnology in oncology, its application in clinical settings still lacks successful results in clinical trials. Therefore, comprehensive preclinical evaluations of various nanocarriers and a thorough understanding of the strategies with which to most effectively target MDSCs to alter the TME effectively are crucial before they can be translated into clinical practice.

## Data Availability

Not applicable.
